# Supplementary material for: Photo-degradation dynamics of five neonicotinoids: Bamboo vinegar as a synergistic agent for improved functional duration
Source: PLoS One. 2019 Oct 17;14(10):e0223708. doi: 10.1371/journal.pone.0223708 (PMC6797178; doi:10.1371/journal.pone.0223708)
Supplement: S1 File — Figs A-J in Supporting Information file summary of the results of extraction ionic and ion fragmentation of neonicotinoids and photo-degradation products. (DOCX) [file pone.0223708.s001.docx]

**Fig A. The extraction ionic and ion fragmentation of imidacloprid.**

**Fig B. The extraction ionic and ion fragmentation of photo-degradation product of imidacloprid.**

**Fig C. The extraction ionic and ion fragmentation of acetamiprid.**

**Fig D. The extraction ionic and ion fragmentation of photo-degradation product of acetamiprid.**

**Fig E. The extraction ionic and ion fragmentation of clothianidin.**

**Fig F. The extraction ionic and ion fragmentation of photo-degradation product of clothianidin.**

**Fig G. The extraction ionic and ion fragmentation of thiamethoxam.**

**Fig H. The extraction ionic and ion fragmentation of photo-degradation product of thiamethoxam.**

**Fig I. The extraction ionic and ion fragmentation of dinotefuran.**

**Fig J. The extraction ionic and ion fragmentation of photo-degradation product of dinotefuran.**
