# Supplementary material for: Photo-degradation dynamics of five neonicotinoids: Bamboo vinegar as a synergistic agent for improved functional duration
Source: PLoS One. 2019 Oct 17;14(10):e0223708. doi: 10.1371/journal.pone.0223708 (PMC6797178; doi:10.1371/journal.pone.0223708)
Supplement: Attachment — Submitted filename: Response to Reviewers.doc [file pone.0223708.s002.doc]

Dear Dr Yulin Gao,

We have revised the manuscript in response to the editor and reviewers’ advice, and the following shows the point by point response to the reviewers’ comments. Additionally, we made some other revisions. All the changes in separate file and labeled ‘Revised Manuscript with Track Changes’ were highlighted in RED.

The language of this manuscript was carefully revised by ‘Elsevier Language Editing’.

We appreciate very much for your time spent on our manuscript and the referees for their valuable suggestions and comments. The revised manuscript has been resubmitted to your journal. We are looking forward to hearing from your final decision when it is made.

Best wishes

Sincerely,

Yongde Yue

22-Aug-2019

**Response to reviewers:**

Reviewer: 1 
1. The typical chromatograms of the photo process including photo-degradation intermediates and starting material of the neonicotinoid should be provided.

Response: We have noticed this problem, and agree with you. The necessary information about typical chromatograms of the photo process has been submitted as ‘Supporting Information’ file.

2. The possible photo-degradation products should be identified and confirmed by comparison with the authentic standards. The possible photo-degradation pathway and product of acetamiprid should be carefully reconsidered.

Response: We have noticed this problem. The photo-degradation product of acetamiprid was identified using LC-Q-TOF-MS with a diode array detector, and which was compared with the results of PhD dissertation ‘Study on Hydrolytic and Photolytic Behavior of Acetamiprid in Environment’ that was completed by Dr. Guohong Xie.
3. Lines 269-270, by separating and identifying the photo-degradation products of the five neonicotinoids in methanol, it can be inferred that the pathways are mainly photo-oxidation processes. Have you separated and identified these photo-degradation products?

Response: We have modified this sentence. We didn’t separated the photo-degradation products, and standards of five neonicotinoids were prepared at 10 mg/L using methanol, then were photo-treated using the high-pressure mercury lamp, and a darkness control was set. Finally, the photo-degradation product samples were detected using LC-Q-TOF-MS.

4. Lines 301-302, it can be observed that the main factors that quench the photo-degradation of neonicotinoids in bamboo vinegar are possibly acetic acid and pigments. To confirm your hypothesis, you’d better to use acetic acid and one pigment to test the quenching effect.

Response: We have noticed this statement, and agree with you. We have modified this sentence. In our study about the photo-degradation of five neonicotinoids with different pH value, in an acidic buffer solution, the photo-degradation rate was low, whereas in neutral and alkaline buffer solutions, the rate was relatively high and generally increased with increasing pH. And then compared with literature which listed as 47, 48, and 69-79 in the manuscript, the hypothesis was obtained. Of course, we will confirm that in the following in-depth study.

Thanks to reviewer’s suggestion, it’s very carefully.

Reviewer: 2

Thank you for such high rating of this manuscript. We have polished and corrected typos and mistakes, and the language of this manuscript was carefully revised by ‘Elsevier Language Editing’. We have modified the conclusions part according the suggestion.

Reviewer: 3

Thank you for such high rating to the results of this manuscript, and we are regret to the quality of the language. Then, we have polished and corrected typos and mistakes, and the language of this manuscript was carefully revised by ‘Elsevier Language Editing’.

Thanks to reviewer’s comments and suggestions.
