# Supplementary material for: Photo-degradation dynamics of five neonicotinoids: Bamboo vinegar as a synergistic agent for improved functional duration
Source: PLoS One. 2019 Oct 17;14(10):e0223708. doi: 10.1371/journal.pone.0223708 (PMC6797178; doi:10.1371/journal.pone.0223708)
Supplement: Attachment — Submitted filename: Response to Reviewers.docx [file pone.0223708.s003.docx]

Dear Dr Yulin Gao,

We have revised the manuscript in response to the editor and reviewers’ advice, and the following shows the point by point response to the reviewers’ comments. Additionally, we made some other revisions. All the changes in separate file and labeled ‘Revised Manuscript with Track Changes’ were highlighted.

The language of this manuscript was carefully revised by ‘AJE Language Editing’ again.

We appreciate very much for your time spent on our manuscript and the referees for their valuable suggestions and comments. The revised manuscript has been resubmitted to your journal. We are looking forward to hearing from your final decision when it is made.

Best wishes

Sincerely,

Yongde Yue

15-Sep-2019

**Response to reviewers:**

Reviewer #3: Your manuscript submission is interesting and contains information that our readers would find useful. AND also this revision looks more better than before, Unfortunately, the quality of the language is not up to the standards of the journal. language error still can be see in the current version.

I suggest you may wish to consider having your paper professionally edited for English language by a native English speaker and/or a professional language editing service here before resubmitting this manuscript：

http://www.zn16289208.icoc.bz/

http://www.unlecture.com/

http://www.goodenglish.com/

http://www.journalexpert.com/

which specialise in editing scientific and technical research papers written in English by authors who are not native English speakers.

Please note that while this service will greatly improve the readability of your paper, it does not guarantee acceptance of your paper by the journal.

Thanks to reviewer’s suggestion, it’s very carefully.

Response: Thank you for such high rating to the results of this manuscript, and we are regret to the quality of the language. Then, we have polished and corrected typos and mistakes, and the language of this manuscript was carefully revised by ‘AJE Language Editing’ according your suggestion.

Thanks to reviewer’s comments and suggestions.
