# Supplementary material for: Photo-degradation dynamics of five neonicotinoids: Bamboo vinegar as a synergistic agent for improved functional duration
Source: PLoS One. 2019 Oct 17;14(10):e0223708. doi: 10.1371/journal.pone.0223708 (PMC6797178; doi:10.1371/journal.pone.0223708)
Supplement: Attachment — Submitted filename: Response to Editor.docx [file pone.0223708.s004.docx]

Dear Dr Yulin Gao,

We have revised the manuscript in response to the editor’ advice, and the following shows the point by point response to the editor’ comments. Additionally, we made some other revisions. All the changes in separate file and labeled ‘Revised Manuscript with Track Changes’ were highlighted.

The language of this manuscript was carefully revised and modified by Prof. Rafael Luque.

We appreciate very much for your time spent on our manuscript and the valuable suggestions and comments. The revised manuscript has been resubmitted to your journal. We are looking forward to hearing from your final decision when it is made.

Best wishes

Sincerely,

Yongde Yue

25-Sep-2019

**Response to editor:**

Editor: After careful consideration, we feel that it has merit but does not fully meet PLOS ONE’s publication criteria as it currently stands. Therefore, we invite you to submit a revised version of the manuscript that addresses the points raised during the review process.

Response: Thank you for such high rating to the results of this manuscript, and we are regret to the quality of the language. Then, we have polished and corrected typos and mistakes, and the language of this manuscript was carefully revised and modified by Prof. Rafael Luque.

Thanks to editor’s comments and suggestions.
